# Biogas production from different food waste using small-scale floating-drum-type anaerobic digester

**DOI:** 10.1007/s00449-025-03239-w

**Published:** 2025-10-29

**Authors:** M. Shehata, Y. Elsayed, A. M. I. Mohamed, M. S. Ismail, M. Walker, I. A. Ibrahim

**Affiliations:** 1 Mechanical Power Engineering Department, Faculty of Engineering, Port Said University, Port Said, 42523 Egypt; 2 School of Engineering, University of Hull, Hull, HU6 7RX UK; 3 Faculty of Industry and Energy, East Port Said University of Technology, North Sinai, 45632 Egypt

**Keywords:** Anaerobic digestion, Floating digester, Fish waste, Potato waste, Leftover cooked food

## Abstract

The generation of food waste poses an escalating societal challenge. Anaerobic digestion emerges as a sustainable and eco-friendly method for valorization and disposal. A small-scale floating-drum-type digester was developed, operating in batch mode to harness biogas from three distinct food waste categories. Potato waste, leftover cooked food, and fish waste were utilized as feedstock, maintained at an average temperature of 21 °C for a retention time of 10 days, with cow manure serving as the inoculum source. The advances of the current work are built upon comparing biogas production volume and methane content from mono-anaerobic digestion of these various wastes. Examining cow manure and different substrate samples offers insights into their composition, encompassing total solids, C/N ratio, and pH. Shredded raw wastes were wet fed into the digester at a 1:1 waste/water ratio. Cumulative production of biogas and the methane fraction from two experiments were monitored. The maximum average cumulative biogas production per kg of total solid was observed for leftover cooked food (up to 261.4 L/kgTS), followed by fish waste (up to 248.5 L/kgTS) and potato waste (up to 137.15 L/kgTS). The maximum methane percentage occurred in fish waste displaying the highest methane percentage (74%), trailed by leftover cooked food (59%) and potato waste (55.8%) from both experiments.

## Introduction

The global sustainable development strategy recommends energy diversification to guarantee a sustainable energy supply and social stability. According to the United Nations Environment Program (UNEP) food waste index report in 2021, about 931 Mt was wasted in restaurants, stores, and other public places in 2019 [1]. A significant portion of the waste is disposed into canals, rivers, or open areas without treatment or even incineration, causing water, soil, and air pollution and disrupting the landscape. Statistics from the National Center for Social and Criminological Research (NCSCR) show that the average amount of food wasted per person in Egypt each year is roughly 91 kg and about 50% from the total amount of fruits and vegetables, 40% of fish, 30% of milk, and 10% of wheat are wasted. It is estimated that only sixty percent of the biomass waste produced in Egypt is collected, of which less than twenty percent is recycled or properly disposed [2]. This threatens public and animal health, making waste-to-energy (WTE) technology essential for improved sanitation and renewable energy. Diverting organic waste from landfills reduces greenhouse gases and pollution, driving interest in alternative energy solutions [3]. Biogas production is a renewable, low-carbon energy resource for the rural communities that currently suffer from inadequate biowaste treatment and unreliable electricity provision.

Anaerobic digestion (AD) technology is an important tool in converting organic waste into a low-carbon energy source: biogas. This process involves the biodegradation and stabilization of complex organic matter by a group of bacteria in the absence of oxygen, producing biogas that can be utilized for various purposes, including electricity generation, cooking, and provision of space heating [4]. Biogas mainly consists of (50–75%) methane (CH_4_) and (25–50%) carbon dioxide (CO_2_) in addition to some trace gases depending on the types and quantities of the supplied feedstock. It has a calorific value of between 26 and 30 MJ/m^3^, depending on the percentage of methane in the biogas [5, 6].

Leftover cooked food (LCF) is widely utilized as feedstock in anaerobic digestion due to its high calorific value, nutrients, and versatile biodegradability. Kuo et al. [7] found that using food waste instead of biosolids, such as sewage sludge, would triple the methane production potential. The study of Cho et al. [8] on different types of food waste (cooked meat, boiled rice and fresh cabbage) revealed that the methane yield is different at the same temperature and retention time owing to the different degradability, water content, organic loading rate, and chemical composition of the materials. According to their findings, substrate properties have a significant impact on methane production, and consistent procedure parameters might not work as well for various kinds of food waste. This emphasizes how anaerobic digestion systems must be optimized for waste. Several studies [9–13] have investigated anaerobic digestion of food waste, with particular emphasis on comparing mesophilic and thermophilic conditions. Agrahari and Tiwari [14] compared different ratios of kitchen waste-to-water using an aluminum biogas plant. The maximum biogas production obtained was about 0.26 m^3^ from 8 kg of kitchen waste at a waste-to-water ratio of (1:2) with a 48% maximum methane fraction. With other ratios of 1:1 and 1:1.4, there was no fraction of methane in the biogas produced. Alam et al. [15] investigated dry anaerobic digestion (DAD) of food waste using three lab-scale single-stage batch digesters. The study employed two distinct feedstock preparation methods; a homogenized mixture of sorted food waste amended with curd (yielding no biogas), versus unprocessed food waste co-digested with livestock manure using mechanical mixing. Only the second approach produced measurable biogas (2079 mL), with methane content verified by combustion quality (blue flame). The results demonstrate the critical importance of feedstock preparation and co-substrate selection in dry anaerobic digestion systems. Basumatary et al. [16] examined how the proportion of feedstock to water (F/W) and temperature influenced biogas production from cattle dung and vegetable waste. Different mixtures of F/W were tested over 55 days to determine the optimal ratio for biogas generation. The highest biogas output was achieved with 60:40 F/W for both materials. The impact of temperature on biogas production was then investigated under 60:40 F/W conditions. Both cattle dung and vegetable waste produced significantly more biogas when processed at mesophilic and thermophilic temperatures compared to traditional methods. However, these findings are constrained by their batch-scale experimental conditions (2L reactors). The necessity for precise temperature control and its associated energy demands pose significant operational and economic barriers that may limit scalability to continuous, full-scale systems.

Potato waste (PW) and fish waste (FW) are also posing a significant environmental challenge due to their contribution to environmental pollution, necessitating the adoption of sustainable solutions to mitigate their impact. Egypt generates roughly 7 million tons of potatoes annually, with an estimated 17% lost as waste across the supply chain—mainly during post-harvest handling, storage, processing, and transportation. This amounts to approximately 1.19 million tons of PW each year [17].

The AD strategies explored by Achinas et al.[18]—particularly their optimized pretreatment and co-digestion approaches—could offer a viable solution for valorizing this underutilized resource. Their demonstrated 9–17% increase in methane yield from potato peel waste (PPW) suggests significant potential for scaling similar AD systems to address both Egypt’s organic waste surplus and energy needs. Liang and McDonald [19] investigated the biogas production from the anaerobic digestion of pre-fermented PPW and showed that a biogas containing about 60–70% methane could be obtained after 8–10 days.

A substantial portion of Egypt’s fish production undergoes processing before reaching the consumer market, with about 70% of the total output being processed [20]. This processing generates significant volumes of fish waste, which typically range between 20 and 80% of the raw fish weight, depending on the processing method (gutting, scaling, filleting) and species-specific characteristics. For instance, finfish processing generates approximately 10–50% non-edible residues (heads, guts, skin, bones, and residual flesh), while shellfish and crustaceans may yield up to 85% non-edible waste (shells, viscera, appendages). Considering Egypt’s recent annual fish production of 492,300 tons [20, 21], the potential annual fish waste generated could be in the range of 98,000 to 394,000 tons, underscoring the scale of the disposal challenge.

However, this significant output also leads to substantial fish waste (FW), which poses challenges for sustainable disposal. Addressing this issue, Xu, Mustafa et al. [22] demonstrated that anaerobic digestion (AD) of FW can be optimized through an inoculum-to-substrate (I/S) ratio of 2.19 and bagasse co-digestion. While their approach enhanced methane yields and process stability, trade-offs like marginally reduced total biogas at higher bagasse ratios highlight the need for context-specific solutions to valorize Egypt’s FW potential effectively. Bücker et al. [23] evaluated the relationship between the microbial community and the biogas production during the anaerobic digestion of two waste types, FW and fish crude oil waste. From their study, they found that the produced methane yield is higher by about 30% with fish waste compared to fish oil. This shows the great potential of fish residues as an alternative substitute for biogas production in mono-digestion processes. Yulisa et al. [24] investigated the effect of substrate-to-inoculum ratio and temperature during start-up period of anaerobic digestion of fish waste. They studied the increase of the ratio from 0.5 to 3, on improving the methane production rate; however, as the ratio increased beyond 1, the methane yield decreased by as much as 21.7% and 39% at 35 °C and 45 °C, respectively. This decline was attributed to an imbalance between the processes of methanogenesis and acidification, which is caused by increased organic loading and the higher temperature affecting the degradation rate of the substrate.

Among the available digester designs, the floating drum type offers several advantages, including simplicity, ease of handling different substrates, and direct monitoring of biogas production volume via displacement measurement of the floating drum. Its relatively low construction cost and user-friendly operation make it suitable for household-scale applications [25], unlike more complex systems such as fixed-dome or plug-flow digesters. However, floating-drum digesters have a limited lifespan, lower capacity, and require a consistent feed source [26, 27]. Despite these limitations, their practicality in decentralized and small-scale contexts makes them an appropriate choice for the current study.

Previous studies on anaerobic digestion of food waste primarily relied on lab-scale reactors with controlled heating systems, limiting their real-world applicability due to high energy demands and scalability challenges. To address these constraints, the present study introduces a small-scale, household-compatible digester designed for practical deployment without external heating. By operating at ambient temperature, the system eliminates the need for energy-intensive temperature control while maintaining efficient biogas production. The novelty of this study lies in its multifaceted approach towards enhancing biogas production through mono-digestion of various organic waste materials. By focusing on the comparative analysis of these waste substrates in a controlled laboratory setting, using a simplified and efficiently designed floating-drum digester, this research aims to shed light on their individual suitability for anaerobic digestion. Despite, the higher biogas yield from the co-digestion process, anaerobic mono-digestion is characterized by the substrate’s capacity to independently supply the microorganisms required for anaerobic digestion. This approach provides the benefit of reducing operating costs and generating stabilized fertilizer [28]. Mono-digestion system is easier and more flexible in handling wastes, particularly in situations where obtaining a significant amount of waste for co-digestion is difficult.

In the present work, a comparison is made of biogas production and methane content from the mono-anaerobic digestion of LCF, PW, and FW. While prior studies have explored kitchen waste and potato waste (mostly on peels and limited on fully spoiled), as well as fish and fish oil waste, the unique contribution of this work is in directly comparing the results of separate (mono) digestion processes for each waste type in a floating-drum digester, rather than focusing solely on co-digestion scenarios of them.

## Materials and methods

### Feedstock and inoculum

Different food waste types were employed in the current work; LCF, PW, and FW are used as feedstock. Cow manure, as a source of methanogenic bacteria, was fed into the digester as an inoculum to help facilitate the mono-anaerobic digestion process. Therefore, a deep understanding of the operational parameters would enhance the applicability of anaerobic mono-digestion for treating and recovering energy. Cow manure was supplied from a village in the south of Port Said city, and PW, LCF, and FW were provided by local sellers. Specifically, 7 kg of PW, 2 kg of LCF, and 6 kg of FW were used in the current work. The employed LCF is composed of leftover food including rice, pasta, cooked meat, cooked peas, and spinach.

An experimental examination was conducted on a fresh cow manure sample to ascertain the proportion of total solids (TS). The analysis revealed a TS percentage of 16%, determined by drying the sample at 105 °C for 24 h, and the nitrogen–carbon content was assessed using a Thermo Scientific™ FLASH 2000 CHNS Analyzer [25], revealing a C/N ratio of 24. The pH value of the sample was measured using a pH digital meter, CDS 107 series, with the initial pH of the fresh sample recorded as 8.1.

The characteristics of the cow manure and the different feedstocks at the beginning of the digestion are listed in Table 1.

**Table 1:** Characteristics of cow manure and different feedstock

Feedstock	Initial TS (%)	Initial pH value
Cow manure (inoculum)	16	8.1
PW	24.8 ± 1.1	5.18 ± 0.04
LCF	28.85 ± 0.2	4.7 ± 0.28
FW	21 ± 1.4	5.72 ± 0.54

An electrical shredder is used to reduce the size of the food wastes. The shredding process enhances the efficiency of anaerobic digestion and facilitates digestion prior to feedstock loading into the digester tank. Namely, shredding increases the surface area of the waste, making it easier to handle, transport, and process. It also helps in the uniform mixing of different waste types. After shredding, wastes were stored in a freezer at − 5 °C before adding them to the digester until characterization and experimental processing were performed.

### Anaerobic digestion unit and measuring facilities

A small-scale anaerobic digestion system was built at the Department of Mechanical Engineering at Port Said University. The system is a floating-drum-type digester composed of a 230 L fermentation tank and a 137 L floating gas holder where biogas is produced. The unit is made of PVC and equipped with instrumentation for measuring the pressure, pH, temperature, and the volume of the produced biogas.

**Fig. 1:**
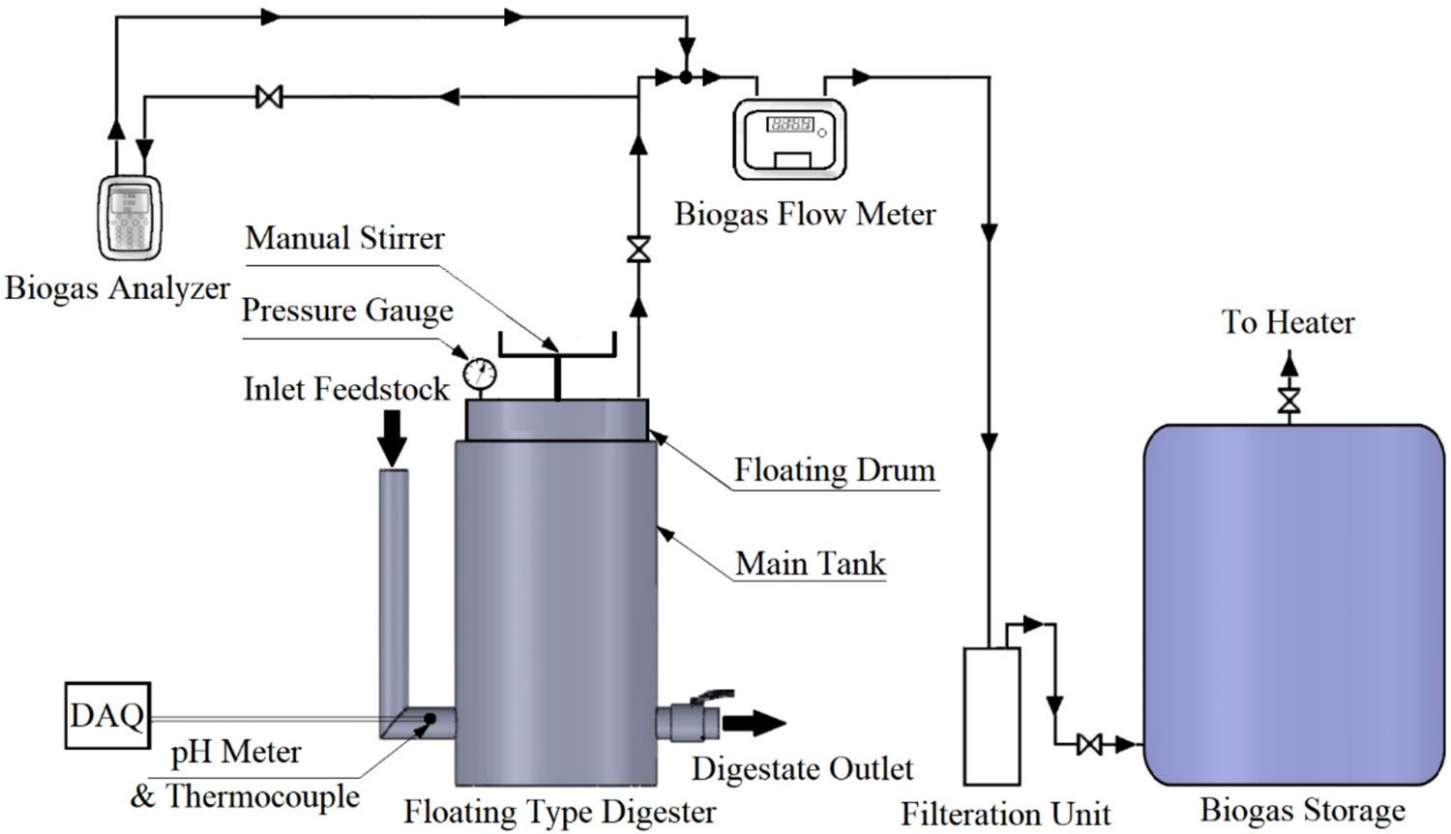
A schematic diagram of the floating-drum-type biogas unit

A schematic diagram of the system is shown in Fig. 1.

**Fig. 2:**
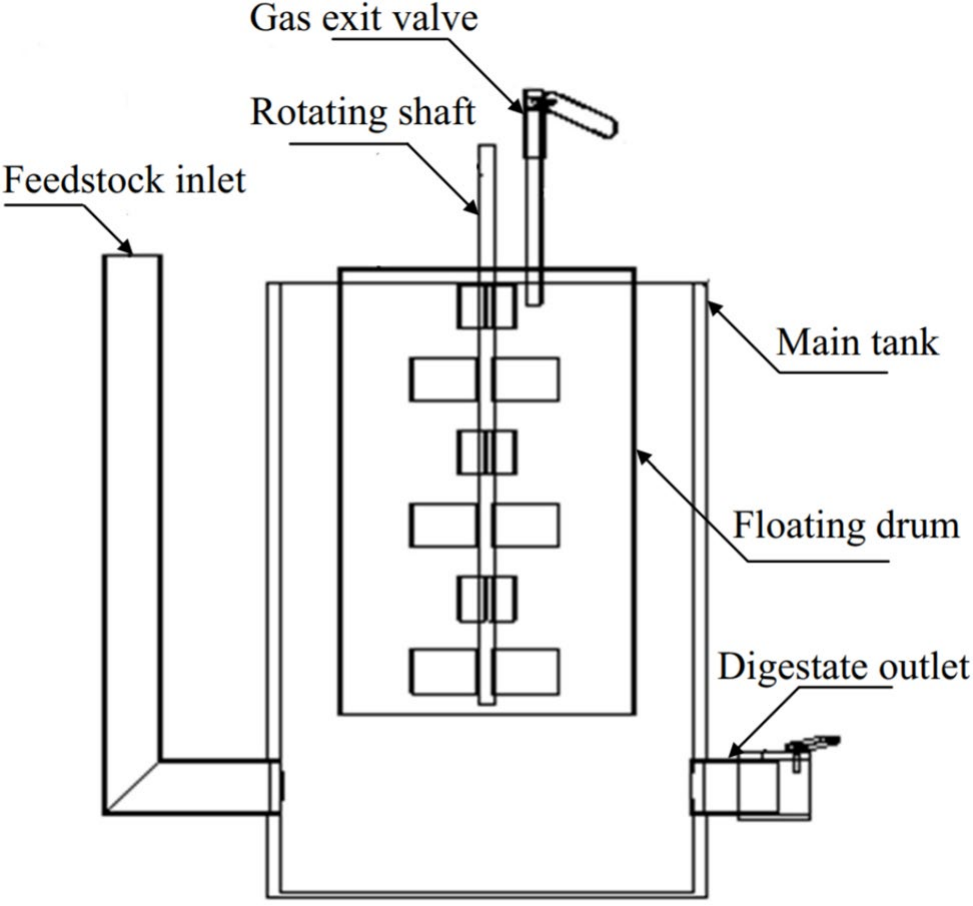
Main components of the developed floating-drum digester

The digestion system is coated in black paint to maximize solar irradiation absorption. Illustrated in Fig. 2, the digester comprises essential components: a main tank for housing all elements and feedstock, an inlet pipe for introducing the waste, a floating gas tank for gas accumulation, and an outlet port mounted atop the floating drum for collecting produced gases. In addition, a rotating shaft works as a manual stirrer for proper mixing within the digester. This shaft measures 80 cm in length, with each blade measuring 7 cm in length, 4 cm in width, and 4 cm in height, arranged in tiers of three blades each, welded at a 45° angle. The gap between consecutive tiers of blades is 15 cm. Processed digestate is extracted from the outlet pipe attached to the main tank.

The experimental work was carried out in moderate conditions when the average temperature was measured using a K-type thermocouple TC-T-1/4NPT-G-120, Omega Engineering, located inside the digester to be 21 °C with measuring range of − 200 to 1350 °C and accuracy of ± 0.75% reading. The digestion was carried out in a batch mode.

First, the digester was loaded with 75 kg of cow manure as an inoculum with 75 L of water to produce the anaerobic microorganisms needed for biogas fermentation. The inoculum has a high population of various bacteria required for anaerobic degradation [25]. The digester gas holder is equipped with a manual stirrer to mix the slurry inside the digester to provide homogeneity. The stirring is made flexible; it occurs once or a few times a day to ensure there is no poor mixing area in the digester and also to break any formation of scum layer on the surface. The target hydraulic retention time was set at 6 weeks until the unit started to produce biogas.

Once the biogas production from the manure reached the minimum yield after the 15th week, different wastes were fed to the digester. The biogas flow rate was measured using a BF-2000 residential biogas flowmeter with an accuracy of <  ± 1.5% and an error percentage in CH_4_ reading of less than 5%. A biogas analyzer device (BIOGAS 5000, Geotech) is used to measure the volumetric percentage of the production biogas components (CH_4_, CO_2_, O_2_, and balance gases) with an accuracy of ± 0.5%.

### Statistical analysis

Data processing, including calculation of means and standard deviations, was conducted using Microsoft Excel 365 and OriginPro 2021 (OriginLab, Northampton, MA, USA). To assess differences in biogas and methane production among the different feedstock treatments (PW, FW, and LCF), a one-way Analysis of Variance (ANOVA) was performed, followed by Tukey’s post hoc test for pairwise comparisons. These analyses were carried out using OriginPro 2021 software to determine statistically significant differences at a significance level of *p* ≤ 0.05.

## Results and discussion

The first phase of the experiments was to investigate the ability to produce biogas in a small-scale digester by employing cow manure as an inoculum. The second phase focused on biogas production from three different food wastes fed into the digester. The volume of produced biogas was daily measured, and a gas analysis was carried out to check the volume fraction of methane in the produced gas. The termination of biogas production was assured before each supply of different waste as the activity of the microorganisms was so weak after each process. This was obvious as the biogas production from cow manure after 15 weeks reduced to less than one L/ day.

**Fig. 3:**
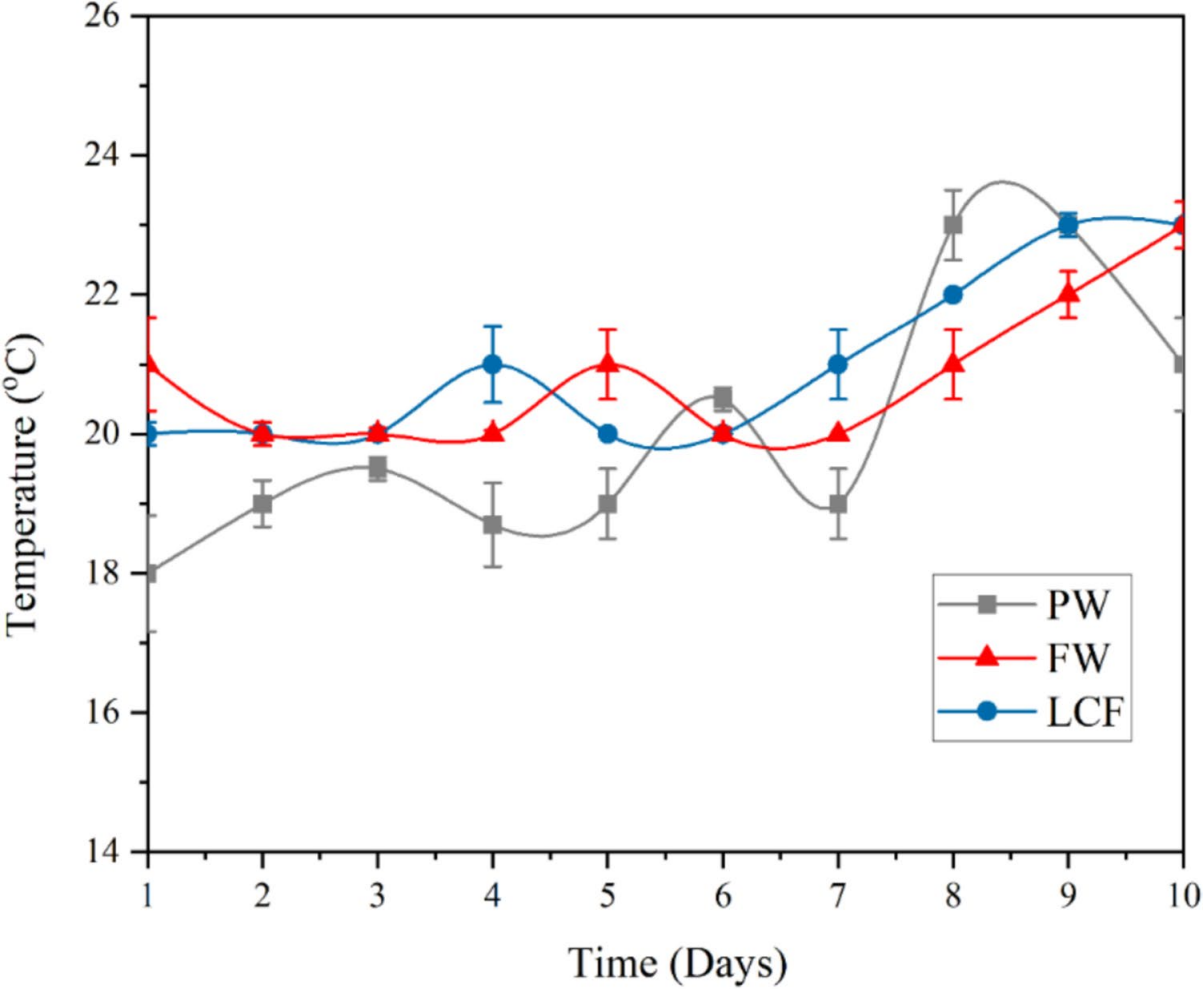
Temperature variation with time for the feedstock in the digester

The experimental trials were conducted between December and May in Port Said, Egypt. During the inoculum phase (December–April), the ambient temperature ranged between 14 °C and 17 °C, while the digestion phase (April–May) experienced temperatures ranging from 18 °C to 23 °C as shown in Fig. 3, with an average of 21 °C. These relatively low values, despite Egypt’s generally hot climate, reflect the seasonal winter-to-spring conditions during the study period.

Solar irradiation was monitored throughout using a calibrated pyranometer, with daily averages ranging from 2.9 to 5.8 kWh/m^2^/day during the winter inoculation phase and 5.8 to 6.4 kWh/m^2^/day during the digestion phase. This environmental data was used to explain the passive heating and performance limits of the digester operating without external temperature control.

**Fig. 4:**
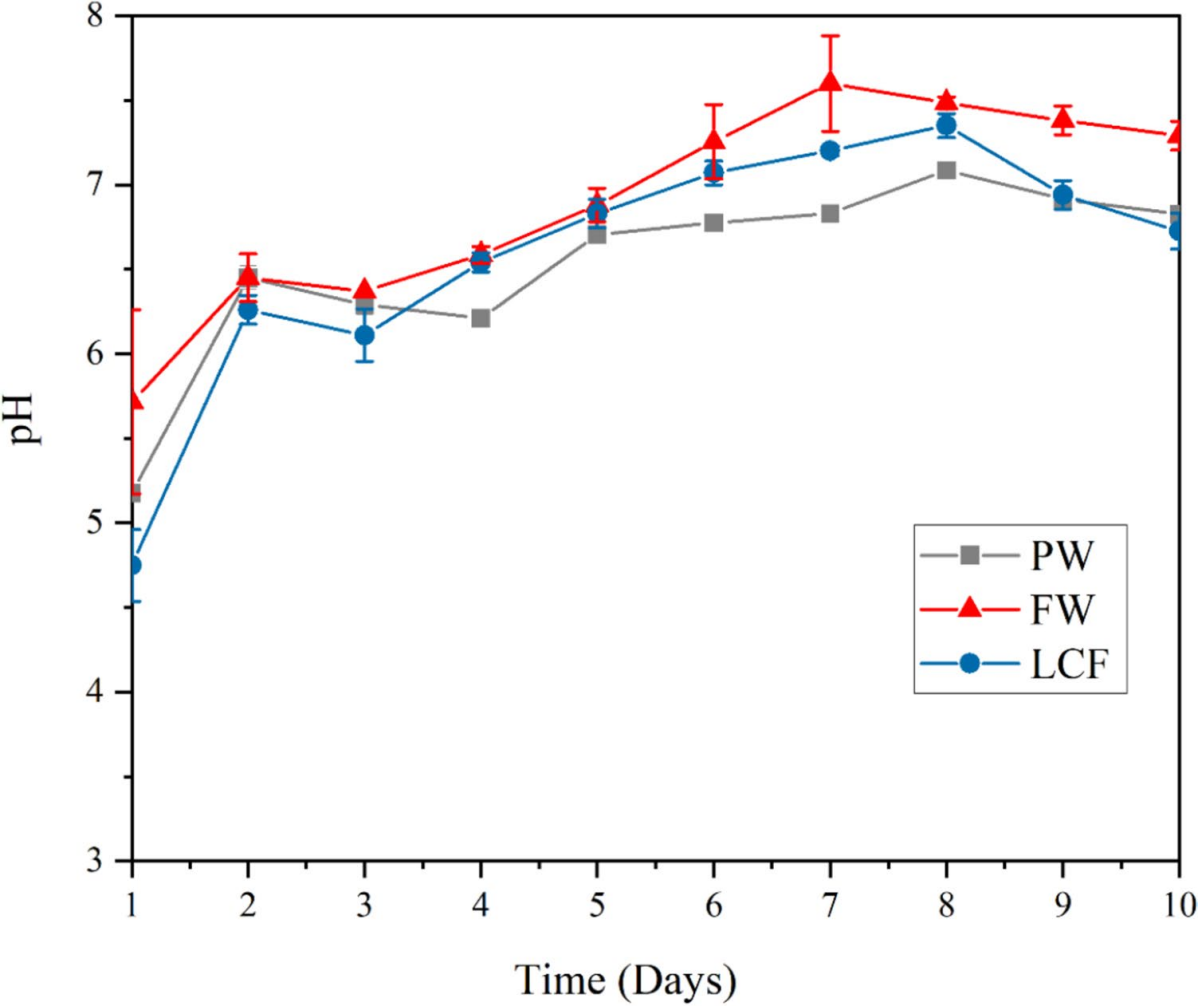
pH variation with time for the feedstock in the digester

In addition to temperature, the pH values of the digester contents were monitored daily throughout the digestion period for each type of feedstock. As shown in Fig. 4, all three waste types followed a similar pH trend, with initial values between 4.5 and 6.1 rising steadily to approach a neutral range (approximately 7.2 to 7.8) by the end of the 10-day period.

### Biogas and methane yield

**Fig. 5:**
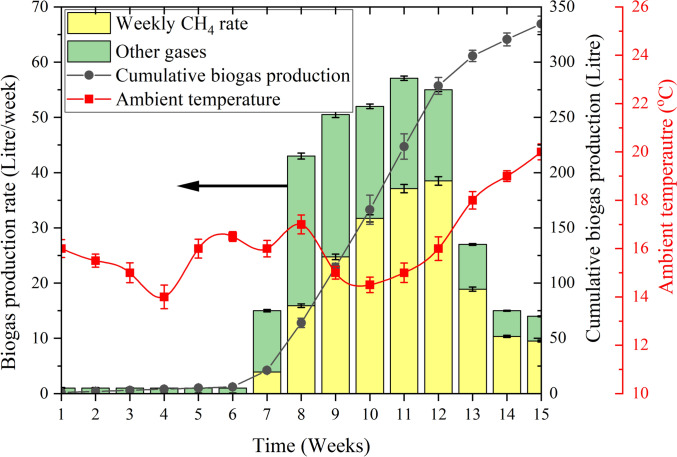
Biogas production from cow manure in 15 weeks

The digester was monitored during the inoculation process for 15 weeks, with a focus on the hydraulic retention time. The maximum gas production was 57 L and observed on week 11, and the cumulative biogas production steadily increased, reaching a total of 334 L by the end of the monitoring period as shown in Fig. 5. The graph also shows the ambient temperature profile, which fluctuated between 13 °C and 22 °C.

These results align with the findings of Paes et al.[29], where cumulative biogas production began at week 7 and reached 10 L/kg after 16 weeks, although the digestion temperature in this study was 24.5 °C. The lower temperature in this experiment likely contributed to the different biogas production rates, as microbial activity in anaerobic digestion is highly sensitive to temperature variations.

Methane (CH_4_) was the dominant component of the biogas from week 8 onwards, contributing substantially to the weekly output.

**Fig. 6:**
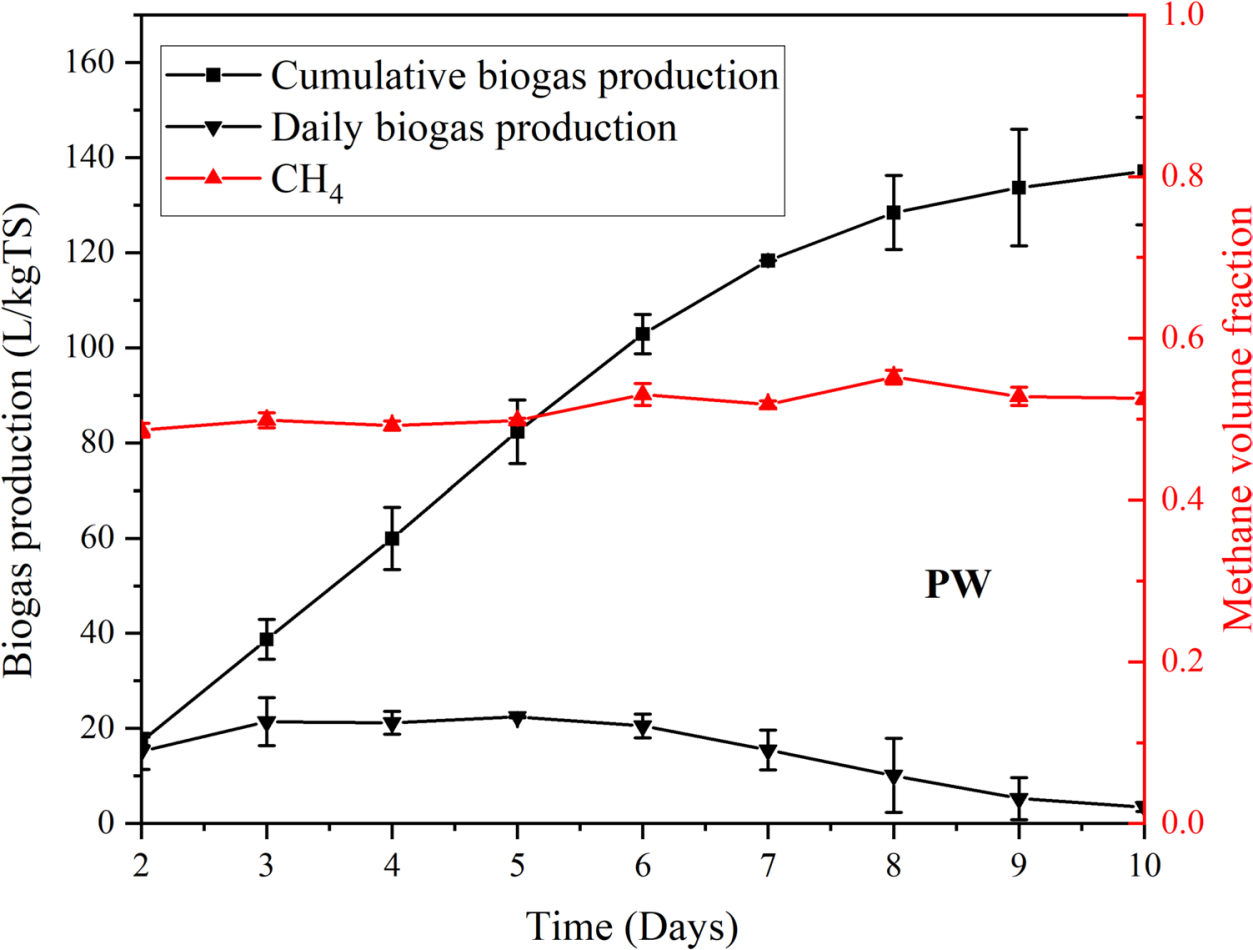
Biogas production (daily and cumulative) and methane volume fraction from PW

After completing biogas production using cow manure, 7 kg of PW were added to the digester. Daily biogas production was recorded at 2:00 pm, yielding a cumulative production of 137.1 L/kgTS over a 10-day period, as depicted in Fig. 6. Methane production began on the second day after PW was introduced and quickly stabilized at around 56% of the biogas volume. Daily biogas production started on the second day, peaked at 22.4 L/kgTS by the fifth day, and then gradually declined to around 3.5 L/kgTS by the 10th day. In comparison with Achinas et al. [18], which reported a biogas yield of 160.5 L/kgTS from potato peels under mesophilic conditions (36 °C) in smaller scale batch reactors (500 mL), the present study obtained a 14.6% lower yield.

**Fig. 7:**
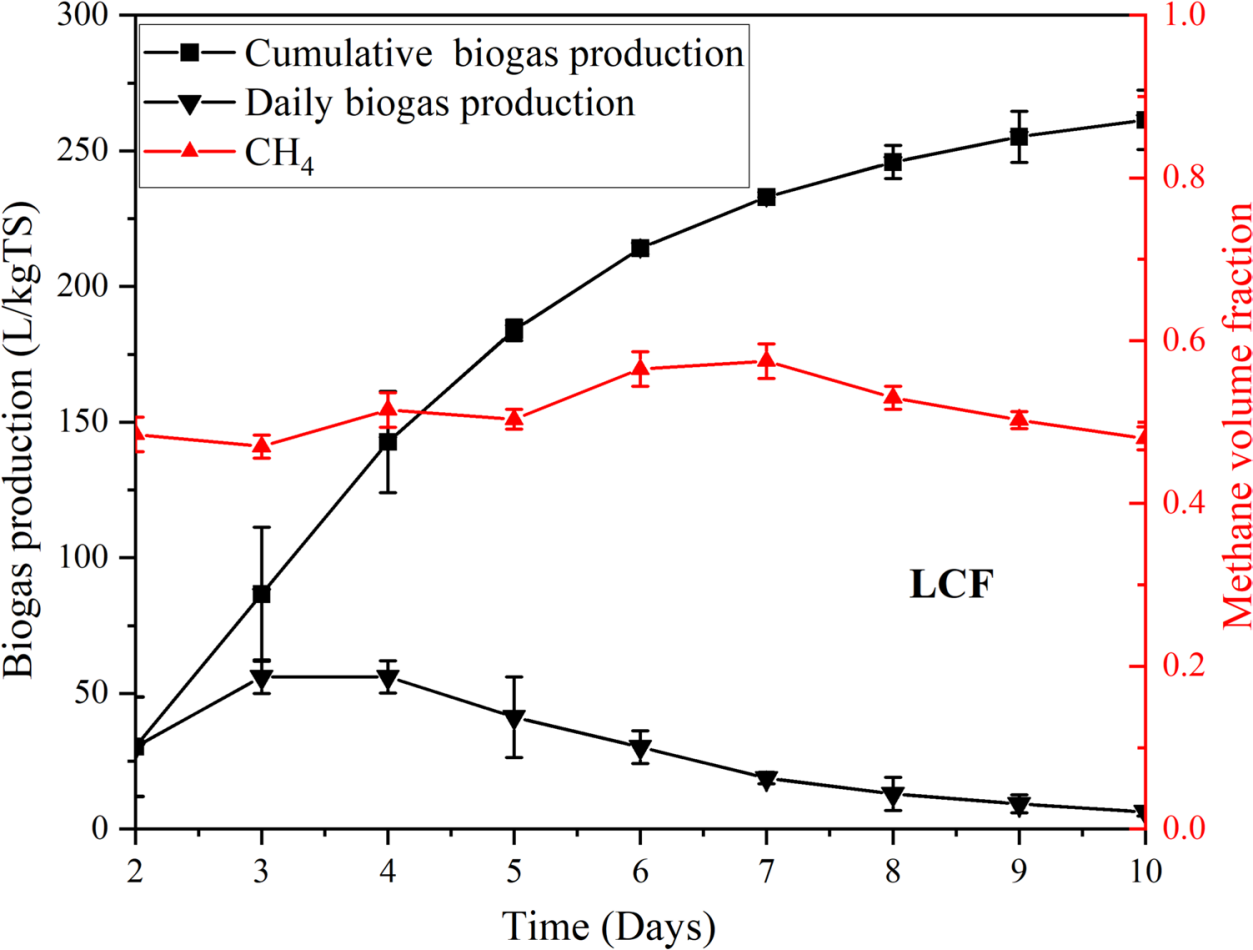
Biogas production (daily and cumulative) and methane volume fraction from LCF

A 2 kg sample of LCF was introduced into the digester. Methane measurements were recorded on the second day to ensure substrate stability and homogeneity, eliminating the influence of prior fermentation processes. The biogas yield was 261.3 L/kgTS, with a methane volume fraction of 50.8%, which was slightly lower than that of PW (52%), as illustrated in Fig. 7. Daily biogas production started on the second day and peaked at 56.12 L/kgTS on the fourth day, and then gradually declined to a minimum. In contrast, the study conducted by Negassa and Turura [30] using various pre-processed and leftover food waste under mesophilic batch conditions achieved a higher specific yield within the same 10-day period, ranging from ≈150 to 360 L/kgTS, depending on feedstock type. This difference is likely attributed to key parameters such as feedstock composition and digester type.

**Fig. 8:**
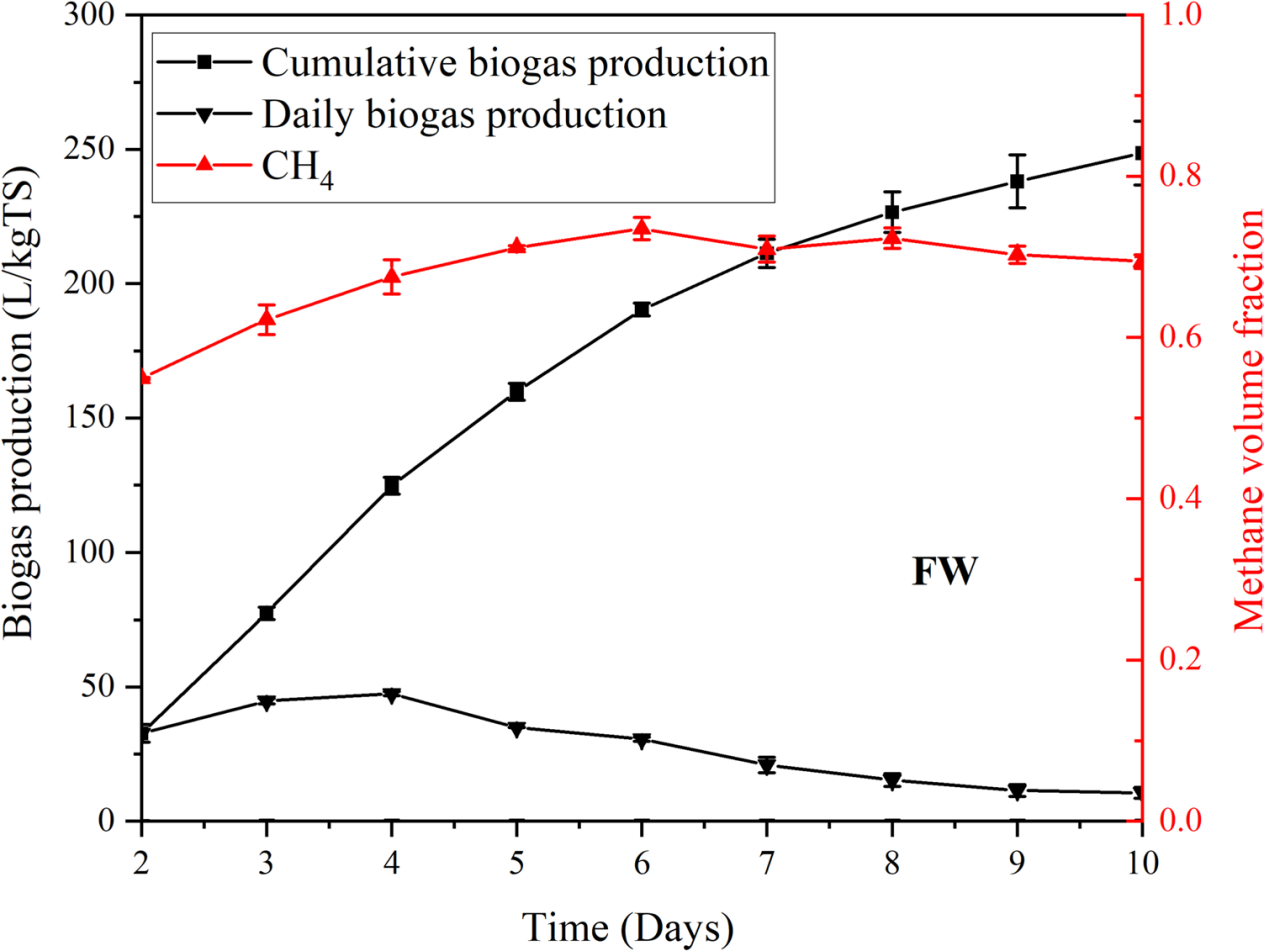
Biogas production (daily and cumulative) and methane volume fraction from FW

A total of 6 kg of FW was fed into the digester; the total biogas production reached 248.5 L/kgTS, as depicted in Fig. 8. The maximum methane volume fraction, approximately 72%, was observed on the seventh day. Daily biogas production began on the second day, peaked at 47.5 L/kgTS on the fourth day, and gradually decreased to its minimum by the end of the 10-day period.

The high production of biogas and methane levels could result from high protein and lipids in FW, which can be easily degraded by microorganisms during anaerobic digestion [23]. In addition, FW may contain a higher concentration of essential nutrients, such as nitrogen and phosphorus, which can stimulate the growth and activity of the microorganisms. This enhances the overall performance of the digester. However, some studies found that the high ammonia level in the digested FW would inhibit digestion later. It was assured by monitoring the pH value of the FW during the process that it was in the optimal range for biogas production [31, 32].

Compared with the study of Xu et al. on FW [22], which reported 406.26 L/kgTS at 37 °C, the present work obtained a 38.8% lower biogas yield under otherwise similar batch conditions. This reduction can be attributed to the lower ambient temperatures used in this study, which possibly slowed down the microbial activity and thus decreased gas production.

### Cumulative biogas volume

**Fig. 9:**
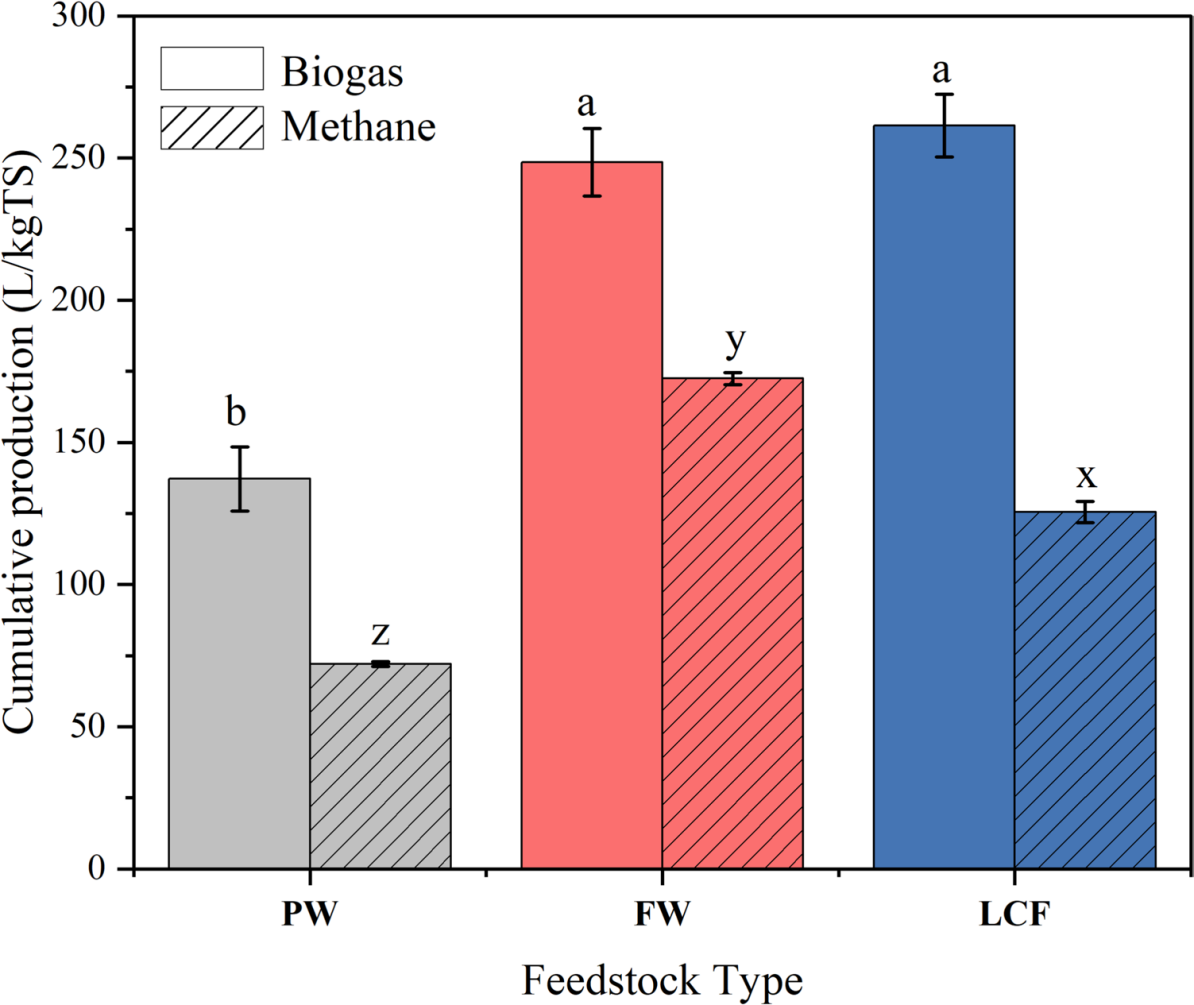
Cumulative biogas and methane yield from different types of waste. Different letters within biogas (a–b) or within methane (x–z) above bars indicate significant differences between feedstocks based on one-way ANOVA followed by Tukey’s post-hoc test (*p* < 0.05)

Figure 9 shows the accumulated biogas produced for each food waste. The volume of biogas produced from the LCF was 261.3 L/kgTS. This was the highest among the other types of waste. Biogas produced from FW was up to 248.5 L/kgTS, while was also up to 137.2 L/kgTS from PW. The balanced carbon-to-nitrogen ratio and high organic content of LCF could elucidate the higher biogas yield; anaerobic microorganisms require a balanced supply of nutrients to efficiently degrade organic matter and produce biogas [33]. The mixture of rice, pasta, meat, peas, and spinach provides a more balanced nutrient composition compared to PW and FW, which may lack certain essential nutrients or have an unbalanced nutrient profile, impacting microbial activity and biogas production.

On the other side, methane production varies notably among the different feedstocks tested. FW feedstock produced the highest cumulative methane yield (172.5 L/kgTS), followed closely by LCF (125.5 L/kgTS), while PW generated the lowest methane volume.

The statistical evaluation was performed separately for both cumulative biogas and methane production using one-way ANOVA across the three feedstocks (PW, FW, and LCF). For biogas, the results showed a significant effect of feedstock type on gas yield (*p* = 0.0029). Tukey’s post hoc comparisons indicated that both FW and LCF yielded significantly higher biogas compared to PW (*p* < 0.01), whereas the difference between FW and LCF was not statistically significant (*p* = 0.56). For methane, the ANOVA also revealed a highly significant difference between treatments (*p* < 0.001). Post hoc comparisons using Tukey’s test showed that all pairwise differences were statistically significant (*p* < 0.005).

### Methane yield of LCF

In this section, a comparison of the anaerobic digestion results of LCF is established between the current work and the experimental work of Das et al. [34]. There are some common points between both studies that make this comparison valid. These points are summarized in Table 2.

**Table 2:** Comparison of the kitchen waste digestion of the current work and Das et al. [34]

Parameter	Current work	Das et al. [34]
Waste type	LCF	Kitchen Waste
Waste-to-water ratio	1:1	1:1
Temperature	ambient	ambient
Digester material	PVC	PVC fiber
Type biogas plant	Floating type	Floating type
Digester volume (L)	230	1000
Mass of feedstock (kg)	2	10
Days of gas production (Day)	10	10
Total biogas production (L/kgWW)	73.5	39.6

**Fig. 10:**
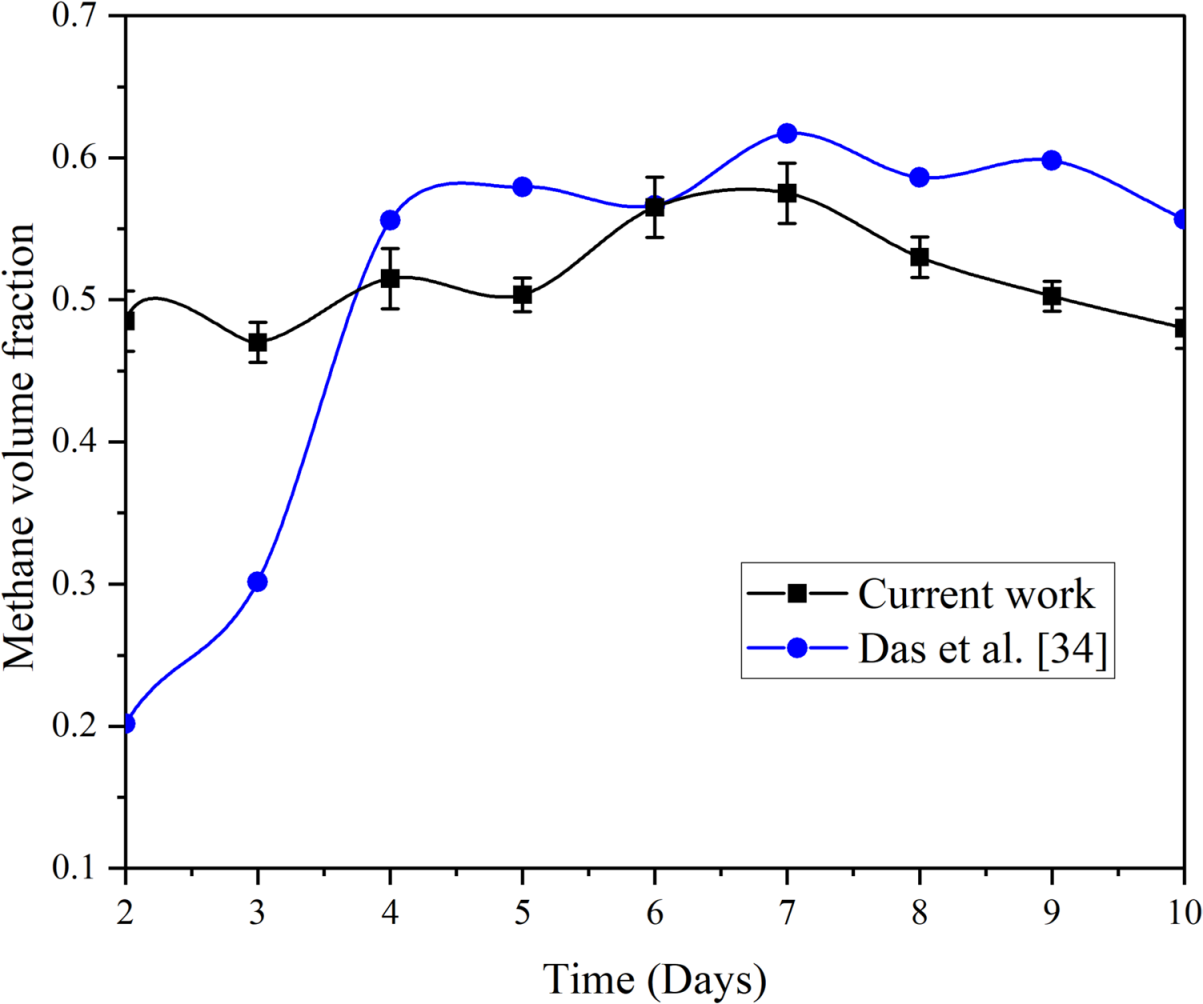
Volume fractions of methane from kitchen waste as reported from Das et al. [34] and the current work

Figure 10 shows that the variation of the methane volume fraction with time in the present work is less compared to that of Das et al. [34]. Notably, the maximum volume fraction of methane was found to be on the 7th day in both studies. The fraction is then reduced beyond the peak value until the end of the duration. As shown in Table 2, the biogas produced from the current study is higher by about 46%. This difference could be due to different parameters, including the composition and the quality of the kitchen waste used. The variations in organic content and nutrient composition, or the presence of inhibitory substances, can influence biogas production. Further, more efficient mixing and improved contact between the microorganisms ensures uniform distribution of microorganisms and nutrients, promoting optimal anaerobic digestion and higher biogas production rates.

Table 3 illustrates a comparative analysis of the biogas yields (L/kg TS) from the present study on PW and FW, and the previous work using different feedstocks (potato peels, fish waste (FW), and fish processing waste). While most prior studies employed small-scale (500 mL) batch digesters at mesophilic temperatures (36–37°C), this work utilizes a larger floating-type digester (230 L) under ambient conditions (21°C). Notably, our system achieved competitive yields (137.15 L/kgTS for PW and 248.5 L/kgTS for FW) despite operating at lower temperatures, highlighting the potential for scaling ambient-temperature AD systems.

**Table 3:** A comparison between the present study and the previous work on PW and FW

Feedstock type	Digester type—scale	Feeding method	Temperature (°C)	Biogas yield (L/kgTS)	Year	References
Potato peels	Glass bottle—500 mL	Batch	36	160.5	2019	[18]
FW	Glass bottle—500 mL	Batch	37	406.26	2017	[22]
Fish processing waste	Glass bottle—500 mL	Batch	37	160.8	2019	[35]
FW	Floating type—230 L	Batch	21	248.5	2025	This study
PW	Floating type—230 L	Batch	21	137.15	2025	This study

These findings indicate that scalable digester designs can generate considerable biogas yields, even in non-thermophilic and uncontrolled ambient conditions. This supports the practicality of low-energy anaerobic digestion systems in real-world applications.

## Conclusion

A study on a 230 L floating-drum digester demonstrated the feasibility of mono-anaerobic digestion of organic wastes like leftover cooked food (LCF), potato waste (PW), and fish waste (FW). LCF produced the highest biogas yield (261.3 L/kgTS), followed by FW and PW, indicating its potential as a superior feedstock for small-scale digesters. However, FW exhibited the highest methane gas content of up to 74%, surpassing that of PW and LCF. The system produced competitive yields compared to previous lab-scale investigations, demonstrating ease of use, scalability, and resilience to changes in feedstock mass and composition. The successful application of a floating-drum digester in rural communities suggests its viability for sustainable organic waste management and renewable energy generation.

## Data Availability

The datasets used or analyzed during the current study are available from the corresponding author upon request.

## Abbreviations

AD: Anaerobic digestion
ANOVA: Analysis of variance
C/N: Carbon/nitrogen ratio
DAD: Dry anaerobic digester
FW: Fish waste
F/W: Feedstock to water ratio
ISR: Inoculum-to-substrate ratios
LCF: Leftover cooked food
Mt: Mega-tons
NCSCR: National Center for Social and Criminological Research
PW: Potato waste
TS: Total solids
UNEP: United Nations Environment Program
VS: Volatile solids
WTE: Waste-to-energy
WW: Wet weight
